# Supplementary material for: Finding positive meaning in memories of negative events adaptively updates memory
Source: Nat Commun. 2021 Nov 15;12:6601. doi: 10.1038/s41467-021-26906-4 (PMC8593143; doi:10.1038/s41467-021-26906-4)
Supplement: Supplementary file 1 — Supplementary Information [file 41467_2021_26906_MOESM1_ESM.pdf]

Finding positive meaning in memories of negative events adaptively updates memory

Megan E. Speer, Sandra Ibrahim, Daniela Schiller, & Mauricio R. Delgado

Exclusions for Experiments 1-3 by Group

Experiment 1 Exclusions	Negative	Positive	Neutral	Distraction	Total
Failed to return for 2nd session	1	0	2	1	4
Computer issues	0	0	0	1	1
Did not recall specific negative memories in 1st session	3	2	5	2	12
Had fewer than 50% of memories that met criteria*	4	5	1	2	12
Total per group:	8	7	8	6	29

Experiment 2 Exclusions	Positive	Control	Total
Failed to return for 2nd session	9	11	20
Failed to return for 3rd session	6	5	11
Did not recall specific negative memories in 1st session	0	2	2
Difficulty using positive meaning finding	4	N/A	4
Total per group:	19	18	37

Experiment 3 Exclusions	Immediate test	Delayed test	No reminder	Total
Failed to return for 2nd/3rd session	2	5	4	11
Adverse weather	0	1	0	1
Computer issues	1	2	0	3
Did not recall specific negative memories in 1st session	1	3	0	4
Had fewer than 50% of memories that met criteria*	4	0	2	6
Difficulty using positive meaning finding	2	1	4	7
Total per group:	10	12	10	32

**This was due to participants not following directions or reporting it was not the same memory across retrievals.*

Supplementary Methods & Results

Experiment 1: Positive meaning finding leads to enhanced positivity at future retrieval

Instructions for each elaboration condition:

Positive group: "Please focus on the positive aspects of this memory. Describe something you learned from this negative event, something positive that occurred because of it, or how it is meaningful to you in some way."

Negative group: "Please focus on the negative aspects of this memory. Describe why you view this memory negatively, something negative that occurred because of it, or what details about this memory make it negative to you."

Neutral group: "Please focus on the neutral aspects of this memory. Describe when and where this event took place (e.g., date and location)."

Coding for written content

Positivity of Content Rating: This was rated in terms of both how positive the event details were as well as the tone of the overall description of the memory. A recollection that only described the negative details of the event and had a negative tone received the lowest rating.

Scale 1-10: Very Negative (1) to Very Positive (10)

Examples of memories rated as low and high in positive content:

Low Positivity	High Positivity
There was a hurricane last summer. After it was over, there were fallen trees throughout my whole neighborhood and a few tall trees in our backyard that I feared would fall on our house. Our basement was flooded and we didn't have electricity for a full week. My younger sister had a really hard time with this, which made me really sad. It was so scary and I wasn't sure what to do about it.	On New Year's Eve I got into a big fight with my cousin about my ex-boyfriend. It seemed awful at the time. But I realize that everything he told me was meant to help me, not make me upset. He told me that my ex-boyfriend wouldn't be the right person for me in the long run. I'm glad someone had the courage to tell me this. It also helped my cousin and I learn how to communicate better.

<u>Content Dissimilarity Rating</u>: This rating was based on changes in event details across retrievals. In particular, coders were asked to rate the degree to which the same (conceptual) event details were present, rather than if the narrative was told in the exact same way or with the exact same words.

Scale 1-10: Very Similar (1) to Very Dissimilar (10)

Example of a memory rated as being high in content dissimilarity across retrievals:

Recall 1	Recall 2
When my family and I went on vacation to West Palm Beach, the first thing we did was go to the pool at our hotel. There were separate kids and adults sections. I thought the kids section was really boring because there were barely any kids there at all. I decided to go to the bigger part of the pool where everyone else was. But I lost hold of the edge of the pool and couldn't keep my head above water and almost drowned. What made it worse was that my mom was talking and drinking at the bar inside the pool, and it took her a long time to come help me.	I went on a family vacation in Florida last year. I almost drowned on the day we arrived. I was trying to play basketball in the pool with my older sisters but it was too deep. It was awful. But afterwards, we spent the rest of the night in our hotel room watching really fun movies. My parents felt really bad about not paying close enough attention to me, so I got to choose what we did the next day. I definitely did not want to go back to the hotel pool for the rest of the trip, so they let us go to the beach instead.

Negative autobiographical memory retrieval

On average, participants spent 2.49 minutes (SD = 1.04) writing descriptions for Recall 1, 1.49 minutes (SD = 0.68) writing elaborations and 2.00 minutes (SD = 0.85) writing descriptions for Recall 2. Recall/elaboration durations (Recall 1 duration: $F_{3,98} = 0.35$, p =.79; Elaboration duration: $F_{3,98} = 0.32$, p = .73; Recall 2 duration: $F_{3,98} = 0.22$, p = .88) and the number of memories used in analyses (M = 9.46, SD = 1.88; $F_{3,98} = 0.27$, p = .85; 21.2% of memories excluded) did not differ by group (*Positive* = 9.35, SD = 1.96; *Negative* = 9.72, SD = 1.67; *Neutral* = 9.28, SD = 2.05; *Distraction* = 9.50, SD = 1.90). There were also no group differences in mood (negative affect: $F_{3,98} = 0.24$, p = .87; positive affect: $F_{3,98} = 0.65$, p = .59) or baseline ratings of feeling ($F_{3,98} = 1.25$, p = .30), intensity ($F_{3,98} = 0.86$, p = .46), vividness ($F_{3,98} = 0.66$, p = .58) or age of the memories ($F_{3,98} = 1.22$, p = .31) during Recall 1, suggesting that participants recalled memories of similar emotional quality and spent a similar amount of time thinking and writing about them, regardless of group assignment. A one-way ANOVA for change in intensity (Recall2 – Recall1) by group was not significant ($F_{3,98} = 0.69$, p = .56).

Experiment 2: Changes in emotion and memory content are long-lasting

Baseline Memory Ratings

There were no group differences in baseline ratings of feeling (t_{89} = -0.466, p = .642), intensity (t_{89} = 0.037, p = .971), vividness (t_{89} = 0.706, p = .482), significance (t_{89} = 1.09, p = .277), social context (t_{89} = -1.56, p = .123), frequency of recall in daily life (t_{89} = 0.774, p = .441) or age of the memories (t_{89} = -0.175, p = .861), suggesting that the *Positive* and *Control* groups recalled memories of similar emotional quality during Recall 1.

Individual differences

Within the *Positive* group we also tested for individual differences that might relate to one's success in changing their memories over time, specifically looking at clinical symptomology (depression, anhedonia). Interestingly, individuals reporting fewer anhedonia symptoms (e.g., loss of pleasure) showed the greatest increase in positive content across retrievals (r_{44} = -.292, p = .049; Fig. S1a), suggesting that experiencing anhedonia may relate to less effective memory updating with positive content. When examining this by each retrieval period separately, these results were primarily driven by the 2-month retrieval (r_{44} = -.309, p = .036; Fig. S1c) although the 1-week retrieval was in the expected direction (r_{44} = -.232, p = .121; Fig S1b).

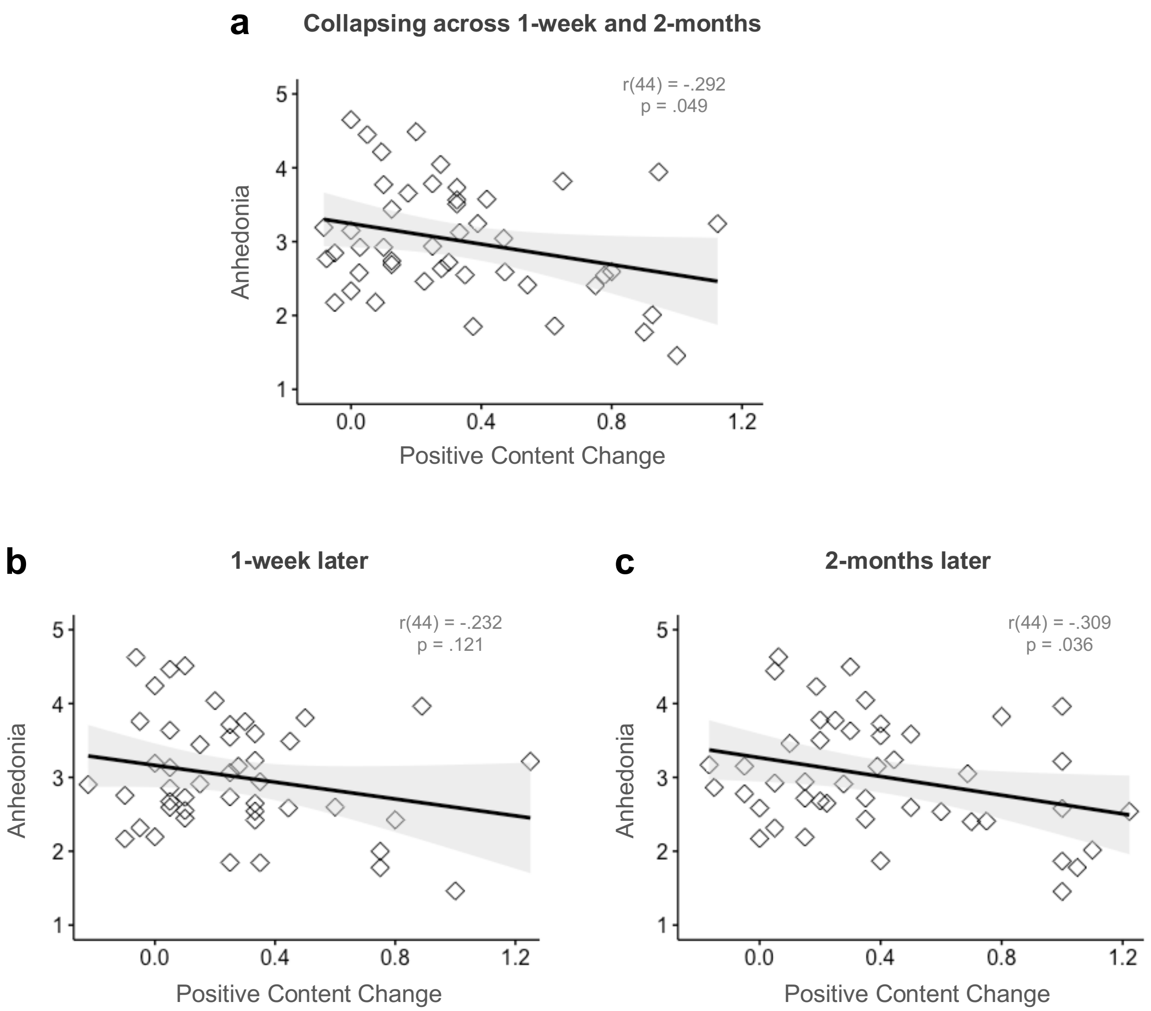

Supplementary Fig 1. Anhedonia negatively correlates with memory content change across time. Within the Positive group (n = 46), greater anhedonia symptomology was associated with a smaller increase in positive content over time: a) collapsing across 1-week and 2-month retrievals; b) 1-week later; c) 2-months later. The shaded band represents the 95% confidence interval on the best-fitting regression line. Source data are provided as a Source Data file.

Experiment 3: Adaptive updating leverages memory reconsolidation mechanisms

Baseline Memory Ratings & Reaction Time

We conducted condition (positive, control) by group (*Delayed-Test, Immediate-Test, No-Reminder*) ANOVAs for each baseline rating of memory separately. We found no significant main effects of condition, group or interactions for baseline intensity ($F_{1,138}$ = 0.001, p = .980; $F_{2,138}$ = 0.83, p = .436; $F_{2,138}$ = 0.15, p = .864), vividness ($F_{1,138}$ = 0.003, p = .959; $F_{2,138}$ = 0.164, p = .849; $F_{2,138}$ = 0.019, p = .982), social context ($F_{1,138}$ = 0.001, p = .979; $F_{2,138}$ = 0.21, p = .808; $F_{2,138}$ = 0.043, p = .958), or age of the memories ($F_{1,138}$ = 0.16, p = .691; $F_{2,138}$ = 2.50, p = .086; $F_{2,138}$ = 0.28, p = .756). We did, however, observe a significant main effect of group for baseline feeling ratings ($F_{2,138}$ = 4.29, p = .016), but no effect of condition ($F_{1,138}$ = 0.01, p = .923) or interaction ($F_{2,138}$ = 0.04, p = .962). When exploring this further, we found that the *No-Reminder* group had significantly greater baseline feeling ratings than the *Immediate-test* group (t_{47} = 2.81, p = .007) while neither group differed from the *Delayed-test* group (t_{45} = 1.30, p = .201; t_{46} = 1.52, p = .137). Therefore, we controlled for baseline feeling ratings in our analyses. In addition, groups did not differ in memory onset or recall duration in any session (all p > .135), This suggests similar ease of retrieval despite methodological timing differences in timing of recall (1h vs. 24h delay).

Experiment 4: Neural pattern dissimilarity is associated with memory change

Changes in emotion across memory retrievals

We examined the degree to which memories changed in their emotional feeling ratings across retrievals from before (Recall 1) to 24h after positive elaboration (Recall 2). In line with our prediction, positively reinterpreted memories elicited enhanced positive emotion at future retrieval (24h later) as compared to memories that were naturally recalled ($t_{31} = 5.13$, $p < .001$, $d = 0.91$). Importantly, memories in these two conditions did not differ in baseline ratings of feeling, intensity, vividness, frequency of recall or age (all $p > .20$), indicating that these factors cannot account for our findings. They also did not differ in baseline onset or recall duration, suggesting that neither condition had memories that were easier or more difficult to remember.

To test the longevity of the effect, we asked participants to return 2-months later to recall these same memories again in a behavioral session. Almost two-thirds of participants returned (n = 18). Interestingly, memories that had been positively reinterpreted 2-months earlier still showed a greater increase in positivity at retrieval than memories that were not ($t_{17} = 3.23$, $p = .005$, $d = 0.76$), suggesting that positive emotion-focused coping did in fact have a long-lasting impact on memory.

Reaction time during recall and elaboration tasks

Participants did not differ in memory onset or durations between positive and control conditions during Recall 1 (Positive: $M_{onset} = 2.80$, SD = 0.90, $M_{dur} = 3.35$, SD = 2.12; Control: $M_{onset} = 2.78$, SD = 0.92, $M_{dur} = 3.31$, SD = 2.14; $t_{31} = .258$, $p = .798$; $t_{31} = .346$, $p = .731$). Participants had longer memory onset and durations in the positive relative to the control condition during the Elaboration task (Positive: $M_{onset} = 3.63$, SD = 1.35, $M_{dur} = 8.35$, SD = 6.60; Control: $M_{onset} = 2.94$, SD = 0.97, $M_{dur} = 6.85$, SD = 5.84; $t_{31} = 3.85$, $p < .001$; $t_{31} = 3.49$, $p = .001$). In Recall 2, there was no difference in memory onset between conditions, and participants thought about memories slightly longer in the control relative to the positive condition (Positive: $M_{onset} = 2.11$, SD = 0.91, $M_{dur} = 2.66$, SD = 2.27; Control: $M_{onset} = 2.25$, SD = 1.00, $M_{dur} = 2.88$, SD = 2.40; $t_{31} = -1.73$, $p = .093$; $t_{31} = -2.13$, $p = .041$).

fMRI Analyses

For the following univariate analyses, the two Recall tasks and the Elaboration task were modeled using a regressor for memory recall during positive trials, a regressor for memory recall during control trials, and a regressor representing missed trials (i.e., unable to reappraise or not the same memory across sessions). To ensure consistency across participants, we used the mean onset and durations of memory recall and elaboration for the memory and elaboration regressors, respectively (M_{recall}= 3.1, SD = 2.1; $M_{elaboration}$ = 7.6, SD = 6.0). Parametric models additionally included a parametric regressor for the degree of feeling rating change across retrievals (Recall2 – Recall1) during memory recall (orthogonalized with respect to the memory regressors) or during elaboration (orthogonalized with respect to the elaboration regressors).

Elaboration Task: Neural responses to positive meaning finding vs. natural recollection

We first performed whole-brain analyses contrasting memories that were positively reinterpreted (positive trials) relative to naturally recalled memories (control trials) in the Elaboration Task. This revealed activation consistent with prior neuroimaging studies examining positive reappraisal in particular[1] and cognitive reappraisal more generally[2,3]. Specially, when positively elaborating on negative memories, there was greater activity in regions previously implicated in reward, such as bilateral ventral striatum, bilateral caudate, and ventromedial prefrontal cortex (VMPFC)[4,5], as well as regions previously implicated in the cognitive control of emotion, such as left ventrolateral prefrontal cortex (VLPFC), left dorsolateral prefrontal cortex (DLPFC) and dorsomedial prefrontal cortex (DMPFC, Fig. S2a)[1,2].

It is important to note that participants only reported emotion ratings during the two retrievals (Recall 1, Recall 2) and not during the Elaboration task. Our reasoning was that participants' subjective ratings after reinterpretation would occur during the modification period and therefore could potentially become embedded in their memory, which could confound their emotion rating at future retrieval (24h later). To examine the efficacy of positive elaboration, in lieu of these ratings, we tested whether the strength of one's behavioral effect (feeling change across retrievals) was associated with greater neural activity in reward-related and cognitive control-related regions during positive elaboration. Consistent with this prediction, a greater increase in positivity across retrievals (Recall2 – Recall1) was associated with greater activity in the

DLPFC (r_{31} = .450, p = .009) and VLPFC (r_{31} = .354, p = .043) during positive elaboration, suggesting that engagement of this neural circuitry was associated with more successful emotion change over time.

Memory Recall 1 & 2: Neural responses to positive meaning finding vs. natural recollection

We also performed a whole-brain positive > control contrast during Recall 1 and Recall 2, which yielded no significant activations. However, our key analysis was to examine neural activity tracking changes in emotion at the second retrieval (24h after modification occurred). Therefore, we performed a positive > control contrast with the additional inclusion of parametric regressors for feeling change (Recall2 – Recall1) on a trial-by-trial basis for both positive and control trials during Recall 2. This parametric contrast during Recall 2 indexing positive emotion revealed a very similar activation map to the positive > control contrast during the Elaboration task. That is, we found greater activity for positively reinterpreted memories relative to naturally recalled memories as a function of increasing positivity in regions associated with reward and positive affect (ventral striatum, caudate, VMPFC) as well as emotion regulation (VLPFC, DLPFC, DMPFC, Fig. S2b). This suggests that negative memories updated with positive content may re-engage the same corticostriatal circuitry they previously engaged during positive elaboration.

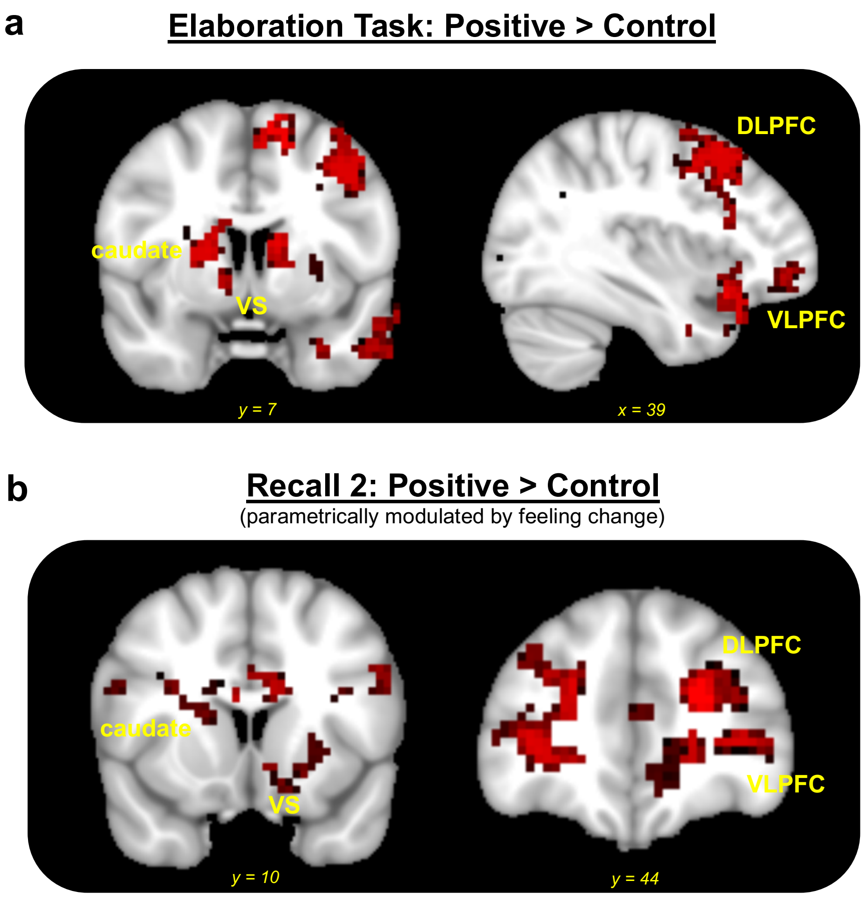

Supplementary Fig 2. Neural responses to positive meaning finding relative to natural recall.

a) Positive meaning finding relative to natural recall during the Elaboration task engaged regions previously associated with subjective value/reward (caudate, ventral striatum) and the cognitive control of emotion (DLPFC, VLPFC; $p < .05$ corrected). b) The same analysis parametrically modulated by positive emotion on a trial-by-trial basis during future retrieval (Recall 2 task) engaged a similar neural circuitry ($p < .05$ corrected).

Exploratory Representational Similarity Analyses (RSA)

Whole-brain Searchlight Analysis

We ran our key RSA analysis as an exploratory whole-brain searchlight RSA analysis using PyMVPA (version 2.6). This analysis performed a searchlight across the brain revealing regions where the correlation between Recall1-Recall2 dissimilarity and increased positivity across time was greater in the positive condition relative to the control condition. We used the same inputs (parameter estimates from a single-trial memory first-level GLM) as the ROI-based RSA analyses. We corrected for multiple comparisons via a non-parametric permutation test (5000 iterations) to reach a corrected alpha < .05. However, this analysis yielded no significant activation after correction.

ROI Analyses

We chose our 3 key ROIs (hippocampus, VS, and VMPFC) based on prior research, such as the hippocampus's key role in memory storage and retrieval[6]. Our prediction was that negative memories could be updated with positive content, but that the negative content may not necessarily lessen or disappear to a degree that we could observe in the brain. Thus, we did not focus our analyses on ROIs typically associated with negative affect, such as the amygdala. Instead we hypothesized that the addition of positive content might be most prominent in reward-related regions (VS and VMPFC) that had been linked to both recalling positive aspects of autobiographical memories in a similar paradigm[7] and when participants use positive meaning finding in response to negative IAPS images in emotion regulation tasks[1].

Given the role of the amygdala in memory and emotion processing, we ran an exploratory RSA analysis focused on this region. We also examined another reward-related region (caudate) that has been linked to these processes. However, when performing the same RSA analysis, neither of these exploratory ROIs showed a relationship between neural pattern dissimilarity and emotion change across retrievals for positive relative to control trials.

We also tested whether neural dissimilarity across retrievals in the positive condition alone was significantly different from zero in our two ROIs that showed a significant difference between positive and control conditions. Neural dissimilarity significantly differed from zero in the hippocampus ($t_{31} = 2.82$, $p = .008$) and is in the expected direction but non-significant in the VS ($t_{31} = 1.54$, $p = .133$). Given the significant

finding for the positive condition in the hippocampus, we also examined this for the control condition. For the control condition alone, neural dissimilarity across retrievals did not significantly differ from zero in the hippocampus (t_{31} = -0.30, p = .765).

RSA: Memory specificity of Recall 1 - Recall 2 pattern change relationship with feeling ratings

We performed an additional analysis to test whether the relationship between neural dissimilarity and feeling change was memory-specific. We first created permuted distributions of this relationship by shuffling the pair assignments (10,000 permutations) of neural dissimilarity and feeling change and then computing our spearman rho correlation for the two ROIs (hippocampus, VS) within each condition and participant separately. We then compared the mean of our observed values with the mean of the permuted distribution across participants for each ROI in the positive condition. Our observed correlation was significantly greater than the permuted distribution in the hippocampus (t_{31} = 2.83, p = .008), suggesting that the relationship between greater neural dissimilarity and greater increases in positivity across time is indeed memory-specific. Although it was in the expected direction, we did not observe this in the VS (t_{31} = 1.53, p = .136).

RSA: Recall 1- Recall 2 without including feeling ratings

If positive elaboration is the only condition that would induce a neural change in memory, then we might expect greater neural dissimilarity in the positive condition relative to the control condition, regardless of emotion change. As an exploratory analysis, we conducted RSA examining Recall 1 - Recall 2 neural dissimilarity between positive and control conditions within our 3 ROIs, without including changes in feeling ratings across time. These analyses revealed no significant differences between conditions in any of our 3 ROIs (Hippocampus: t_{31} = 0.70, p = .488; VS: t_{31} = 1.18, p = .249; VMPFC: t_{31} = 0.48, p = .633). This null result is consistent with the notion that memory is reconstructive in nature, meaning we would expect memories to have some degree of change each time they are retrieved even in absence of an explicit instruction for change, which has been demonstrated in previous fMRI studies[8]. In addition to this, since not all memories change after using positive meaning finding and those that do change, change to varying

degrees, Recall 1 – Recall 2 neural dissimilarity tracking increases in positive emotion might be more meaningful than comparing across positive and control conditions alone.

RSA: Elaboration – Recall 2

A potential hypothesis is that a greater change in the positive condition might lead to greater Elaboration – Recall 2 similarity in the positive condition relative to the control condition. This was not a key analysis because we would have similar predictions for both the positive condition and control conditions (for Elaboration – Recall 2 similarity), making it challenging to meaningfully interpret the results. That is, we would expect high Elaboration – Recall 2 similarity in the control condition because these two periods have the same instructions: "recall the memory naturally." In the positive condition, if our manipulation worked well, we would also expect high Elaboration – Recall 2 similarity. Therefore, it would be difficult to interpret differences in Elaboration – Recall 2 similarity across conditions. Consistent with this, when we ran this exploratory RSA analysis, there was no significant difference between conditions in any of our 3 ROIs (Hippocampus: $t_{31} = 0.24$, $p = .812$; VS: $t_{31} = -0.18$, $p = .856$; VMPFC: $t_{31} = 1.42$, $p = .165$).

Relationship between neural activity during positive elaboration and future memory change

We predicted that neural activity when using positive meaning finding during the Elaboration task might be related to future changes in memory. Therefore, we conducted a whole-brain parametric regression analysis for memories in the positive condition during the Elaboration task weighting each memory by its dissimilarity across retrievals within the hippocampus and the ventral striatum (VS), separately (i.e., correlation distance from RSA analysis). This analysis would yield neural activity associated with greater neural dissimilarity from before to after modification. However, neither of these contrasts revealed activity that survived correction. In addition to examining across the whole-brain, we tested this hypothesis with more targeted analyses. Specifically, we tested for correlation between neural activity during positive elaboration trials in regions associated with reward (striatum, VMPFC) and cognitive control (VLPFC, DLPFC) and neural pattern dissimilarity in the hippocampus and VS on a trial-by-trial basis, separately. However, these analyses showed no significant relationships.

Individual differences related to positive elaboration and future memory change

We were also interested in whether protective factors, such as resilience to stress and ability to savor positive emotions in daily life, might be related to a) neural responses during positive emotion elaboration and b) changes in future memory. We first tested whether these traits were related to greater neural responses in regions associated with positive emotion (striatum, VMPFC) and cognitive regulation (VLPFC, DLPFC) during positive meaning finding (in the Elaboration task). Interestingly, greater savoring ability, as measured by the ERP-Revised (Emotion Regulation Profile-Revised)[9], was related to greater activity in the caudate during positive meaning finding (r_{30} = .483, p = .005; Fig. S3a).

We then tested whether these traits were related to future memory change, such as hippocampal dissimilarity or ventral striatum dissimilarity—both of which significantly differed across the positive and control conditions. We observed a positive correlation that was non-significant but in the expected direction for the hippocampus, when considering pattern dissimilarity across time alone rather than in relation to feeling change. Here, greater savoring ability was associated with greater hippocampal dissimilarity across retrievals between conditions (positive – control trials; r_{30} = .325, p = .069; Fig. S3b). That is, one's natural propensity to savor positive emotions in everyday life is associated with greater neural responses in regions previously implicated in reward or value (i.e., caudate) when positively reinterpreting negative memories. It may also be related to greater changes in the neural representation of memory in the hippocampus over time. There was no relationship between these variables and individual resiliency scores.

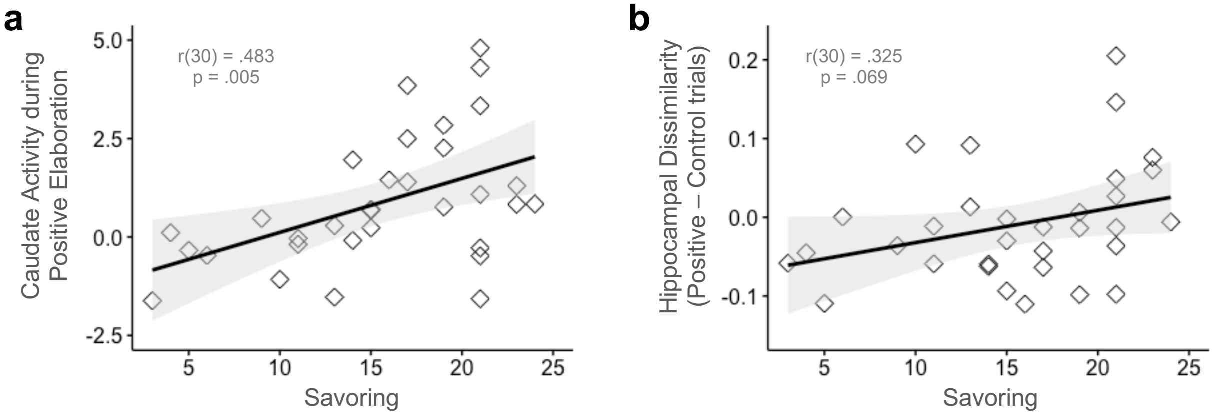

Supplementary Fig 3. Individual differences in savoring relates to neural activity associated with positive meaning finding. A greater ability to savor positive emotions in everyday life was associated with a) greater reward-related activity in the caudate during Positive Elaboration in fMRI scan #1 and b) greater neural pattern dissimilarity in the hippocampus across retrievals (positive – control trials; N = 32 participants). The shaded band represents the 95% confidence interval on the best-fitting regression line. Source data are provided as a Source Data file.

Supplementary Experiment 5: Positive meaning finding vs. monetary reward for memory modification

An outstanding question is whether other manipulations that increase positive emotions would similarly update memory. More specifically, is the generation of positive emotion alone sufficient to update memory, or does the meaningful context of a positive elaboration matter? Thus, this experiment tested whether another positive emotion inducing manipulation—receiving an extrinsic monetary reward—after negative memory retrieval would lead to increased positivity at future retrieval similar to a more internally generated manipulation like positive meaning finding.

This experiment followed a modified version of the *Delayed-test* group in Experiment 3. Fifty-six participants (18 men; M_{age} = 21.5; SD = 6.33) first reactivated 20 negative memories, followed by an elaboration task where they underwent a positive manipulation for 12 memories and naturally recalled 8 memories as a control comparison. For the manipulation trials, the *Positive* group found positive meaning (N=28; 9 men), whereas the *Money* group (N=28; 9 men) had the potential to win a monetary reward ($0.50) during recall. Probabilities were fixed so that participants received money on 12 of the 20 trials, which mirrored the manipulation trials for the *Positive* group. The *Money* group received monetary compensation at the end of the experiment. All participants returned 24h later to test changes in emotion during a second recollection of the same 20 memories.

Methods

Participants

Sixty-nine healthy young adults participated in this 2-day study. Exclusions included failure to return for the second session (N = 5; due to adverse weather, N = 1), computer issues (N = 3), and poor performance on the memory recall tasks (did not recall specific negative memories, N = 3; difficulty using positive meaning finding, N = 2). The final sample included 56 participants (18 men; M_{age} = 21.5; SD = 6.33; 12.5% Asian, 32.1% Black, 30.4% Hispanic,1.8% Pacific Islander, 35.7% White, 5.4% more than one ethnicity) who were randomly assigned to two experimental groups: *Positive Meaning Finding* (N = 28; 9 men) and *Money* (N = 28; 9 men). Using G*Power, we calculated our target sample size to be 56 participants (28 per group) when expecting a medium effect size (80% power). Participants gave informed

consent in accordance with the Rutgers Institutional Review Board for Protection of Human Subjects and received partial course credit and/or monetary compensation for participating.

Experimental Design

This study followed the same design as the *Delayed-test* group in Experiment 3 but was modified to fit into 2 sessions. On Day 1, participants filled out 1) emotion/mood questionnaires, and then completed 2) the AMQ, 3) Recall 1 task, and 4) the Elaboration task. To reduce the potential for fatigue, the Recall and Elaboration tasks only included 20 negative memories (12 positive trials; 8 control trials), instead of 32. In the Elaboration task, the *Positive* group either positively elaborated on the negative memories (positive trials) or recalled naturally (control trials), whereas the *Money* group received $0.50 during negative recall (positive trials) or recalled naturally (control trials). All participants returned 24h later to complete the Recall 2 task, which was identical to Experiment 3 except with only 20 trials.

Data Analysis

We conducted a two-sample t-test examining the differential feeling change across retrievals between manipulation vs. control trials across groups (*Positive, Money*). Significant effects were followed up with post-hoc t-tests using an alpha level of .05.

Results & Discussion

We examined whether receiving an extrinsic reward (e.g., money) after negative memory recall would be effective for positively updating negative memories similar to positive meaning finding. A t-test examining the differential feeling change across time between manipulation and control trials by group was significant (t_{54} = 2.00, p = .051, d = .336). Examining this further, the *Positive* group had a significantly greater increase in positive emotion on manipulation trials relative to control trials (t_{27} = 2.12, p = .044, d = .400), consistent with Experiments 1-4. In contrast, the *Money* group showed no difference between manipulation and control trials (t_{27} = -0.47, p = .639, d = .090), indicating less successful memory updating. This observation highlights how the self-relevant and intrinsically meaningful context of the strategy may

matter, as positive emotion alone (evoked by an extrinsic monetary reward) was less successful in updating negative memories with positive content.

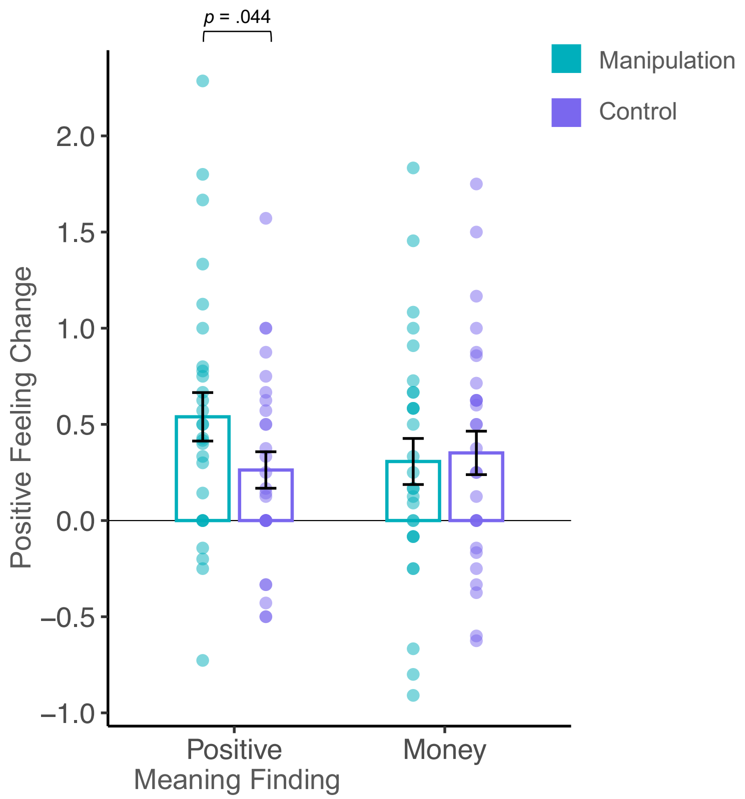

Supplementary Fig 4. Positive meaning finding vs. monetary reward. The Positive Meaning Finding group (n = 28) showed an increase in positive emotion for manipulation relative to control trials (natural recall), whereas there was no difference for the Money group (n = 28), suggesting less successful updating when receiving an extrinsic monetary reward as a potential manipulation for modification. Analyses were two-tailed t-tests. Overlaid dots represent individual participants. Bars show mean values per group and condition; error bars = +/- SEM. Source data are provided as a Source Data file.

Autobiographical Memory Questionnaire Negative Event Cue List

Airport and luggage
Applying for a job
Applying to a school
Arriving late to class
Attending a friend's birthday party
Avoiding someone
Being ignored
Being injured
Being teased
Buying a car
Buying a train ticket
Can't find a parking spot
Cooking an elaborate meal
Dead battery
Delay on morning commute
Doing chores
Eating a new food
Embarrassing yourself
Feeling afraid
Feeling stressed
Flying on a plane
Getting a flat tire
Getting a haircut
Getting caught in the rain
Getting in trouble
Getting locked out
Getting lost
Getting pulled over
Giving a presentation
Going on a family vacation
Going on a mini-road trip
Going on social media
Going through a break-up
Going to a first session of a class
Going to a funeral
Going to a work/club dinner/event
Going to an expensive restaurant
Going to prom / homecoming / school dance
Going to the ATM

Going to the beach
Having a fever
Having an allergic reaction
Having an argument
Having something stolen or being robbed
Hearing about a disaster
Leaving home
Looking for an apartment
Losing an important possession
Loved one moving away
Meeting your advisor
Missing a meeting
Not dressed for the weather
Participating in sports
Passing out
Repainting walls
Running out of gas
Seeing a band in concert
Taking a day trip
Taking an exam
Taking Public Transportation
Telling a lie
Traveling to a nearby state
Traveling to work/school
Visiting a distant relative
Visiting a health professional
Visiting the hospital
Watching the news
Witnessing a car accident

Supplementary References

1. Dore, B. P. *et al.* Finding Positive Meaning in Negative Experiences Engages Ventral Striatal and Ventromedial Prefrontal Reward Regions. *J. Cogn. Neurosci.* (2016).
2. Buhle, J. T. *et al.* Cognitive reappraisal of emotion: A meta-analysis of human neuroimaging studies. *Cereb. Cortex* **24**, 2981–90 (2014).
3. Wager, T. D., Davidson, M. L., Hughes, B. L., Lindquist, M. A. & Ochsner, K. N. Prefrontal-subcortical pathways mediating successful emotion regulation. *Neuron* **59**, 1037–50 (2008).
4. Haber, S. N. & Knutson, B. The reward circuit: Linking primate anatomy and human imaging. *Neuropsychopharmacology* **35**, 4–26 (2010).
5. Delgado, M. R., Nystrom, L. E., Fissell, C., Noll, D. C. & Fiez, J. A. Tracking the hemodynamic responses to reward and punishment in the striatum. *J. Neurophysiol.* **84**, 3072–7 (2000).
6. Chadwick, M. J., Hassabis, D., Weiskopf, N. & Maguire, E. A. Decoding Individual Episodic Memory Traces in the Human Hippocampus. *Curr. Biol.* **20**, 544–547 (2010).
7. Speer, M., Bhanji, J. & Delgado, M. Savoring the past: Positive memories evoke value representations in the striatum. *Neuron* **84**, 847–856 (2014).
8. Xue, G. *et al.* Greater neural pattern similarity across repetitions is associated with better memory. *Science.* **330**, 97–101 (2010).
9. Nelis, D., Quoidbach, J., Hansenne, M. & Mikolajczak, M. Measuring individual differences in emotion regulation: The Emotion Regulation Profile-Revised (ERP-R). *Psychol. Belg.* **51**, 49 (2011).
