# Supplementary material for: Finding positive meaning in memories of negative events adaptively updates memory
Source: Nat Commun. 2021 Nov 15;12:6601. doi: 10.1038/s41467-021-26906-4 (PMC8593143; doi:10.1038/s41467-021-26906-4)
Supplement: Supplementary file 3 — Reporting Summary [file 41467_2021_26906_MOESM3_ESM.pdf]

nature research

Corresponding author(s): Megan Speer & Mauricio Delgado

Last updated by author(s): Oct 20, 2021

# Reporting Summary

Nature Research wishes to improve the reproducibility of the work that we publish. This form provides structure for consistency and transparency in reporting. For further information on Nature Research policies, see Authors & Referees and the Editorial Policy Checklist.

## Statistics

For all statistical analyses, confirm that the following items are present in the figure legend, table legend, main text, or Methods section.

| n/a | Confirmed | |
|---|---|---|
| ☐ | ☒ | The exact sample size ($n$) for each experimental group/condition, given as a discrete number and unit of measurement |
| ☐ | ☒ | A statement on whether measurements were taken from distinct samples or whether the same sample was measured repeatedly |
| ☐ | ☒ | The statistical test(s) used AND whether they are one- or two-sided<br>*Only common tests should be described solely by name; describe more complex techniques in the Methods section.* |
| ☐ | ☒ | A description of all covariates tested |
| ☐ | ☒ | A description of any assumptions or corrections, such as tests of normality and adjustment for multiple comparisons |
| ☐ | ☒ | A full description of the statistical parameters including central tendency (e.g. means) or other basic estimates (e.g. regression coefficient) AND variation (e.g. standard deviation) or associated estimates of uncertainty (e.g. confidence intervals) |
| ☐ | ☒ | For null hypothesis testing, the test statistic (e.g. $F$, $t$, $r$) with confidence intervals, effect sizes, degrees of freedom and $P$ value noted<br>*Give P values as exact values whenever suitable.* |
| ☒ | ☐ | For Bayesian analysis, information on the choice of priors and Markov chain Monte Carlo settings |
| ☒ | ☐ | For hierarchical and complex designs, identification of the appropriate level for tests and full reporting of outcomes |
| ☐ | ☒ | Estimates of effect sizes (e.g. Cohen's $d$, Pearson's $r$), indicating how they were calculated |

*Our web collection on statistics for biologists contains articles on many of the points above.*

## Software and code

Policy information about availability of computer code

| | |
|---|---|
| Data collection | Data was collected using Eprime 2.0 software. |
| Data analysis | Data was analyzed using R (version 3.6), SPM 12, FSL 6.0, Python 3 and PYMVPA (version 2.6). |

For manuscripts utilizing custom algorithms or software that are central to the research but not yet described in published literature, software must be made available to editors/reviewers. We strongly encourage code deposition in a community repository (e.g. GitHub). See the Nature Research guidelines for submitting code & software for further information.

## Data

Policy information about availability of data

All manuscripts must include a data availability statement. This statement should provide the following information, where applicable:

- Accession codes, unique identifiers, or web links for publicly available datasets
- A list of figures that have associated raw data
- A description of any restrictions on data availability

The behavioral data and code that support the findings of these studies are available on OSF (https://osf.io/jtgfk/). We are unable to share written memory content because it includes identifiable information. Source data are provided with this paper.

# Field-specific reporting

Please select the one below that is the best fit for your research. If you are not sure, read the appropriate sections before making your selection.

☐ Life sciences ☒ Behavioural & social sciences ☐ Ecological, evolutionary & environmental sciences

# Behavioural & social sciences study design

All studies must disclose on these points even when the disclosure is negative.

| | |
|---|---|
| Study description | We used a quantitative experimental design, with four experimental groups in Experiment 1, two experimental groups in Experiment 2, three experimental groups with a within-subject factor in Experiment 3, and one experimental group with a within-subject factor in Experiment 4 (fMRI). |
| Research sample | Participants were healthy undergraduate students from Rutgers University and the surrounding Newark, NJ area. Experiment 1 included 102 participants (35 men; Mean age= 20.3; SD= 2.9; 16.7% Asian, 22.5% Black, 23.5% Hispanic, 2.0% Pacific Islander, 41.2% White, 2.0% more than one race) across the Negative (N=25; 9 men), Positive (N=26; 8 men), Neutral (N=25; 8 men), and Distraction groups (N=26; 10 men).<br><br>Experiment 2 included 91 participants (39 men; Mean age= 20.9; SD= 3.89) who were randomly assigned to two experimental groups: Positive (N=46; 20 men) and Control (N=45; 19 men).<br><br>Experiment 3 included 72 participants (29 men; Mean age= 22.3; SD= 6.54; 6.4% Asian, 31.9% Black, 26.4% Hispanic, 23.7% White) across the Immediate-test (N=25; 10 men), Delayed-test (N=23; 10 men), and No-reminder groups (N=24; 9 men).<br><br>Experiment 4 (fMRI) included 32 participants (12 men; Mean age= 22.8; SD= 4.67; 37.5% Asian, 21.9% Black, 18.8% Hispanic, 34.4% White). |
| Sampling strategy | We used G*Power to determine sample sizes. For Experiment 1, our target sample size was 100 participants (25 per group) expecting a medium effect size (80% power). For Experiment 2, our target sample size was calculated to be 90 participants (45 per group) expecting a small effect size (for detecting differences in written memory content as indicated in Experiment 1; 80% power). For Experiment 3, we expected a large effect size (based on Experiment 1 results for emotion change across groups), which yielded a target sample size of 75 participants (25 per group; 80% power). For Experiment 4, our target sample size was 35 participants based on prior fMRI studies using similar multivariate analyses and behavioral data from Experiment 3. |
| Data collection | All data was collected on a computer. Participants were alone while completing tasks and questionnaires. Researchers who conducted the study were aware of experimental conditions, because each condition had aspects of the study with different instructions, but were blind to each study's hypotheses. |
| Timing | Data collection began in February 2016 and ended in December 2018. |
| Data exclusions | Experiment 1: Exclusions included failure to return for the second session (N=4), computer issues (N=1), those who did not recall specific negative memories (N=12), or had fewer than 50% of memories that met criteria (N=12).<br><br>Experiment 2: Exclusions included failure to complete the second (N=20) or third session (N=11) or poor performance on the memory recall tasks (did not recall specific negative memories, N=2; difficulty using positive meaning finding, N=4).<br><br>Experiment 3: Exclusions included failure to return for the second or third session (N=11, N=1; due to adverse weather), computer issues (N=3), and poor performance on the memory recall tasks (did not recall specific negative memories, N=4; remembered <50% of memories from Day 1, N=6; difficulty using positive meaning finding, N=7).<br><br>Experiment 4: Exclusions included poor performance on the memory recall tasks (difficulty using positive meaning finding, N=4; remembering <50% of memories across sessions, N=1) and motion >3mm in any direction (N=3). |
| Non-participation | Experiment 1: Four participants failed to return for the second session.<br>Experiment 2: Thirty-one participants failed to complete the second or third session.<br>Experiment 3: Twelve participants failed to return for the second or third session (N=1 due to adverse weather).<br>Experiment 4: All participants completed the initial 3 sessions (including the two fMRI scans). Fourteen participants failed to return for the 2-month behavioral follow-up session. |
| Randomization | Participants were randomly assigned to groups for all studies. |

# Reporting for specific materials, systems and methods

We require information from authors about some types of materials, experimental systems and methods used in many studies. Here, indicate whether each material, system or method listed is relevant to your study. If you are not sure if a list item applies to your research, read the appropriate section before selecting a response.

## Materials & experimental systems

| n/a | Involved in the study | |
|---|---|---|
| ☒ | ☐ | Antibodies |
| ☒ | ☐ | Eukaryotic cell lines |
| ☒ | ☐ | Palaeontology |
| ☒ | ☐ | Animals and other organisms |
| ☐ | ☒ | Human research participants |
| ☒ | ☐ | Clinical data |

## Methods

| n/a | Involved in the study | |
|---|---|---|
| ☒ | ☐ | ChIP-seq |
| ☒ | ☐ | Flow cytometry |
| ☐ | ☒ | MRI-based neuroimaging |

# Human research participants

Policy information about studies involving human research participants

| | |
|---|---|
| Population characteristics | See above. |
| Recruitment | Participants were recruited using a combination of local flyers and online recruitment tools at Rutgers University (e.g., Sona). |
| Ethics oversight | Rutgers Institutional Review Board for Protection of Human Subjects |

Note that full information on the approval of the study protocol must also be provided in the manuscript.

# Magnetic resonance imaging

## Experimental design

| | |
|---|---|
| Design type | Event-related tasks |
| Design specifications | The Recall 1 and Recall 2 task both included 1 block of 24 trials. Each trial included 14s memory recall, self-paced emotion ratings, and a jittered 6-10s ITI. The Elaboration task included 24 trials across 4 blocks. Each trial included 20s elaboration, a self-paced rating, and a jittered 6-10s ITI. |
| Behavioral performance measures | During Recall 1 and Recall 2 tasks, we recorded recall onset and duration during the memory recollection window (14s) and emotion ratings, both based on participants' button presses. During the Elaboration task, we collected elaboration onset and duration during the elaboration window (20s) based on participants' button presses. We also asked participants to report whether they were successful in using the instructed strategy, which was used to determine whether that trial remained in the analysis. |

## Acquisition

| | |
|---|---|
| Imaging type(s) | Functional |
| Field strength | 3 Tesla |
| Sequence & imaging parameters | We collected neuroimaging data using a 3T Siemens Magnetom Trio scanner. We acquired structural images using a T1-weighted MPRAGE sequence in 176 1mm sagittal slices (256 x 256 matrix, FOV = 256mm) and functional images in 35 contiguous oblique-axial slices (3 x 3 x 3mm voxels) set parallel to the AC-PC plane with a single shot gradient echo EPI sequence (TR = 2s, TE = 25ms, FOV = 192, flip angle 90, bandwidth = 2,232 Hz/Px, echo spacing = 0.51). |
| Area of acquisition | A whole brain scan was used. |

Diffusion MRI ☐ Used ☒ Not used

## Preprocessing

| | |
|---|---|
| Preprocessing software | Images were preprocessed using SPM 12. We motion-corrected each time series to its first volume, and then performed spatial unwarping to minimize geometric distortions due to susceptibility artifacts. We coregistered the mean functional image to the anatomical scan, normalized the anatomical using the unified segmentation model, and then resliced the functional data to Montreal Neurological Institute (MNI) standard stereotaxic space. We then applied spatial smoothing using a Gaussian kernel of 5mm FWHM. |
| Normalization | We coregistered the mean functional image to the anatomical scan, normalized the anatomical using the unified segmentation model, and then resliced the functional data to Montreal Neurological Institute (MNI) standard stereotaxic space. |
| Normalization template | Montreal Neurological Institute (MNI) |
| Noise and artifact removal | To minimize the impact of head motion, we applied additional preprocessing steps using FSL 6.0. Specifically, we detected motion spikes using the FSL tool fsl_motion_outliers. This tool evaluates motion spikes with two metrics: 1) |

root-mean-square (RMS) intensity difference of each volume relative to the reference volume from the first time point; and 2) frame-wise displacements, which are the mean RMS change in rotation/translation parameters relative to the reference volume. We labeled volumes as spikes using a boxplot threshold (75th percentile plus 1.5 times the interquartile range) for metric values within a run, and then removed these spikes via regression. Across all participants, this removed 10% of volumes (range: 3.3 to 19.5%). We then extracted brain material from the functional images and normalized the entire 4D dataset using a single scaling factor (grand-mean intensity scaling). To remove low frequency drift in the MR signal, we applied a high-pass temporal filter (100-s cutoff).

Volume censoring

See above.

## Statistical modeling & inference

Model type and settings

We conducted representational similarity analyses (RSA) to examine neural pattern similarity between the two retrieval sessions (Recall 1 and Recall 2) as a function of condition (positive, control). To conduct RSA, we first computed a GLM modeling each memory as a single regressor for each recall period (Recall 1, Recall 2) and participant separately. We then extracted the multivariate neural pattern in each ROI for each memory in both recall tasks for each participant. After, we calculated the correlation distance between the two neural patterns of activity for Recall 1 and Recall 2 of the same memory in a particular ROI, which made up the RDM for brain space. We then constructed a similar RDM for feature space, which included the corresponding emotion change values for each pairwise comparison. To test our hypothesis that positive meaning finding leads to greater neural dissimilarity (than natural recall) across retrieval sessions as a function of increasing positivity, we computed RDMs separately for positive and control trials. Then, we calculated the spearman rho correlation between RDMs for brain space (in a particular ROI) with RDMs for feature space (change in feeling rating across retrievals) for each participant and for each condition, separately. Finally, we compared the mean correlation coefficients across conditions (at the group level) using a one-sample sign permutation test (5000 iterations) on the difference in rho values between conditions using the nltools python toolbox.

Effect(s) tested

We compared the mean correlation coefficients across conditions (at the group level) using a paired t-test (corrected via a non-parametric permutation test with 5000 iterations).

Specify type of analysis: ☐ Whole brain ☒ ROI-based ☐ Both

Anatomical location(s)

Anatomical locations were determined using a neurosynth parcellation.

Statistic type for inference
(See Eklund et al. 2016)

See below.

Correction

To correct for multiple comparisons, we used a non-parametric permutation test (5000 iterations) to obtain an alpha<.05.

## Models & analysis

| n/a | Involved in the study |
|---|---|
| ☒ | ☐ Functional and/or effective connectivity |
| ☒ | ☐ Graph analysis |
| ☒ | ☐ Multivariate modeling or predictive analysis |
